# Advice to Gonorrheal Patients

**Published:** 1899-11

**Authors:** W. L. Champion

**Affiliations:** Atlanta, Ga.


### ADVICE TO GONORRHEAL PATIENTS.*

By W. L. CHAMPION, M.D.,

ATLANTA, GA.

I have a very short paper, with a time-worn subject, which I wish to present, hoping it will bring forth a full and free discussion, benefiting us all.

By the laity gonorrhea has always been considered a very insignificant trouble, easily cured without any serious after effects. Because the disease does not directly produce death a large percentage of the members of the profession are prone to view the trouble in the same light.

When we consider the serious train of troubles that result from gonorrhea, such as stricture, involvement of the testicles, bladder and kidneys, rheumatism, the large number of people in the blind

* Read before the Atlanta Society of Medicine October 5, 1899.

asylums directly due to gonorrheal ophthalmia, and the pelvic diseases in women due to gonorrheal infection, it impresses me that the profession should awaken to the important fact that the laity should be informed, not only of the highly infectious nature of the disease, but the number of invalids produced by such infection. We can in a measure accomplish this result by teaching our patients not to contract the disease, its serious nature, to go to the physician for treatment, not the druggist, and that there should be no question that the disease is cured before sexual intercourse. Now, some may say that advice of this kind to a patient is worthless, but the physician can and does have some influence over his patrons, and good advice in this direction will be worth something to humanity. We know the disease will always be with us, but we can and should strive to suppress it in some degree.

The question has been asked—Is gonorrhea ever cured? Does the urethra ever return to its normal condition after an attack of the disease? The fact that gonococci remain dormant in the urethra for years would make the above question pertinent. I recently had a patient (married man) to come for treatment who had a urethral stricture which was due to a case of gonorrhea contracted fifteen years before. There was no discharge from urethra and urine was very nearly clear of clap-shreds. A sound was passed into the bladder and the next day there was a profuse purulent discharge, which was examined microscopically and gonococci found in abundance. Of course we cannot believe the statement of every patient with a venereal disease, but this is only one case of quite a number that I could cite where gonococci remained in the urethra for years. That the gonococcus is the cause of gonorrhea there can be no doubt, and it is a fact that gonococci do remain in the urethra for a long period of time, and though they may be inactive, any slight irritant or a decreased resistance of the tissues to the poison will cause a fresh outbreak of the disease. In my opinion, what is generally termed a "bastard clap" is an acute exacerbation of a chronic condition. We do see, though very rarely, cases of non-specific urethritis due to irritating discharges, strong injections, improper use of instruments or highly acid urine, and not due to the gonococcus. Now we can determine positively that the gonococcus is not present when we set up a discharge in the urethra and the examination shows a negative result. That the urethra does return to its normal condition after a specific infection, as does the lung after pneumonia, can be demonstrated in over fifty per cent. of cases taken in time and properly treated. That this is true can be shown by careful examination with the bulbous bougie or ocular inspection through the urethroscope. A urethra that is badly strictured never returns to its former anatomical condition from either medical or surgical treatment, but a badly strictured urethra can by proper treatment be enabled to perform its functions satisfactorily, freed from gonococci, will not shorten life and without the probability of recontracting.

Years ago, and to a large extent at the present time, the acknowledged course to pursue in the treatment of an acute "clap" was to give some mild saline draught until the acute symptoms subsided and then commence active treatment. It is just as reasonable to wait for acute symptoms to subside before commencing treatment in gonorrheal ophthalmia as in gonorrheal urethritis. The foundation for a urethral stricture is formed in the acute stage of the disease, and in proportion to the intensity of the inflammation is the liability to stricture following. The proper course to pursue, is to commence treatment as soon as the patient presents himself and the disease is cut short while we are waiting for the acute symptoms to subside.

It is always the physician's duty to look to his patient's interest and give him the best treatment possible. That better results are obtained by having the patient report to the office daily for treatment instead of giving him medicine to use, will not be questioned by any one who has given it a trial. I will not go into the treatment, as I read a paper before this Society in January, 1898, on The Treatment of Gonorrhea by Hydrostatic Irrigatien.

The physician should, by all means strongly impress upon the patient the highly infectious nature of the disease, also the frequency with which the disease is apparently cured only to break out afresh, and the very important point of avoiding sexual intercourse until entirely cured. A test as to whether a urethra is inflamed, and one that should always be made, is too frequently overlooked, that is, the presence of clap-shreds in the urine. I never dismiss a gonorrheal patient until the urine is free of these shreds.

A physician is very neglectful of his duty if he dismisses a gonorrheal patient without carefully examining his urethra for stricture. The inflammatory area around a stricture is a hot-bed for gonococci, and all that is necessary to arouse them from their dormant state is a slight irritant. When a patient has had a recent gonorrhea or there is any indication of an imflammatory condition of the urethra, a solution of nitrate of silver should be thrown into the urethra to set up a discharge so it can be examined for the gonococcus. This should by all means be done when the patient contemplates marriage. I feel safe in stating that every one of us in this hall to-night would be surprised to know the number of innocent wives infected by their husbands who have an apparently cured specific urethritis. If Eve was truly the cause of Adam's eating the forbidden fruit, womankind has since suffered sufficiently on account of man's ignorance or "cussedness" in infecting woman.

It is true that the majority of men look upon a discharge of pus from the urethra as pathological, but a urethra may contain gonococci when the morning drop is not present. As far as the presence of pus is concerned, this does not hold good with women, and as Prior has said, "we should persuade them to look upon pus from the genitals as having the same significance as pus from other localities." My experience has been that 95 per cent. of male patients with a purulent discharge from the urethra, no matter how slight the discharge, nor whether due to a stricture or not, contains gonococci. As already stated, after throwing a strong solution of silver nitrate into the urethra and examining the discharge produced without finding the gonococcus, is a safe test that the canal is not infected. Still, this will not hold in every case unless we examine carefully to determine whether the small glands and follicles are still involved. To show the importance of a careful examination in every case I will mention one I now have on hand. A young man contemplating marriage came for treatment. He had a chronic urethritis; examination showed discharge replete with gonococci. After six weeks' treatment endoscope showed healthy condition, urine free from clap-shreds, discharge produced by silver nitrate examined and gonococci absent. A few weeks later this patient presented himself with a folliculitis, a small abscess beside the frenum with no inflammation of urethra. Pus from this abscess was examined and gonococci found. In this case there was a single point of infection in a small gland which no doubt the wife would have been infected from should the patient have married. This case illustrates the importance of a careful examination in every respect before dismissing a patient.

While these apparenily little facts are probably known to all of you, we occasionally, in the hurry of business, overlook them. If I can influence some member of the profession to treat his patients, not allow the patients to treat themselves, see them daily with the necessary examination, impress upon them the untold misery they will produce by infecting others, and the importance of knowing they are well before treatment is stopped, I will feel I have accomplished something.

*701, 702 and 703 Prudential Bldg.*

